# Supplementary material for: Cyclophilin D plays a critical role in the survival of senescent cells
Source: EMBO J. 2024 Oct 24;43(23):11. doi: 10.1038/s44318-024-00259-2 (PMC11612481; doi:10.1038/s44318-024-00259-2)
Supplement: Supplementary file 1 — Appendix [file 44318_2024_259_MOESM1_ESM.pdf]

Appendix for

Cyclophilin D plays a critical role in the survival of senescent cells

Table of contents: Page

Appendix Figure S1 2

Appendix Figure S1

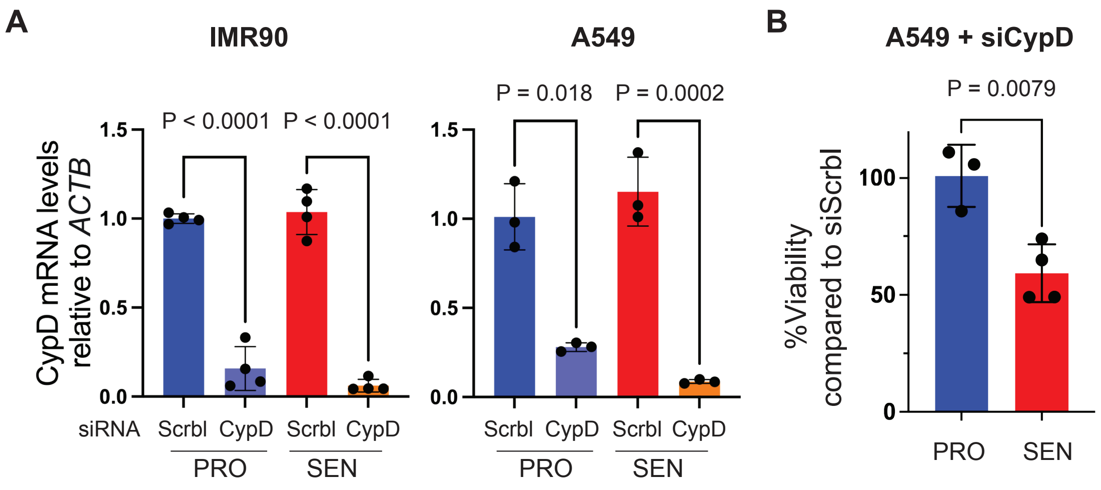

Appendix Figure S1: **Reduced senescent cell viability after CypD depletion assessed by crystal violet staining.** Related to Figure 2.

A Relative mRNA expression of CypD in proliferating (blue) or senescent (red) IMR90 and A549 cells, treated with siRNA Scrbl or siRNA against CypD for 7 days. Signals were normalized to that of β-actin. n = 3-4 biologically independent samples. All the values plotted in the graphs are the mean ± SD. Statistical analyses were performed with 2-way ANOVA multiple comparison with Tukey's correction. P-values are indicated in the figure.

B Proliferating and senescent A549 cells were treated for 7 days with siRNA Scrbl or anti-CypD and stained with crystal violet solution for 10 min. The absorbance was measured at 540 nm following the dissolution of the dye. n=3 (PRO) and 4 (SEN) independent experiments. The values plotted in the graphs are the mean ± SD. Statistical analyses were performed with Unpaired Student's t-test. P-values are indicated in the figure.
