# Supplementary material for: Cyclophilin D plays a critical role in the survival of senescent cells
Source: EMBO J. 2024 Oct 24;43(23):11. doi: 10.1038/s44318-024-00259-2 (PMC11612481; doi:10.1038/s44318-024-00259-2)
Supplement: Supplementary file 4 — Source data Fig. 2 [file 44318_2024_259_MOESM4_ESM.zip › SD Figure 2/2A_WBs.docx]

**Figure 2 - Panel A**

IMR90

A549
