# Supplementary material for: Cyclophilin D plays a critical role in the survival of senescent cells
Source: EMBO J. 2024 Oct 24;43(23):11. doi: 10.1038/s44318-024-00259-2 (PMC11612481; doi:10.1038/s44318-024-00259-2)
Supplement: Supplementary file 9 — Expanded View Figures [file 44318_2024_259_MOESM9_ESM.pdf]

Expanded View Figures

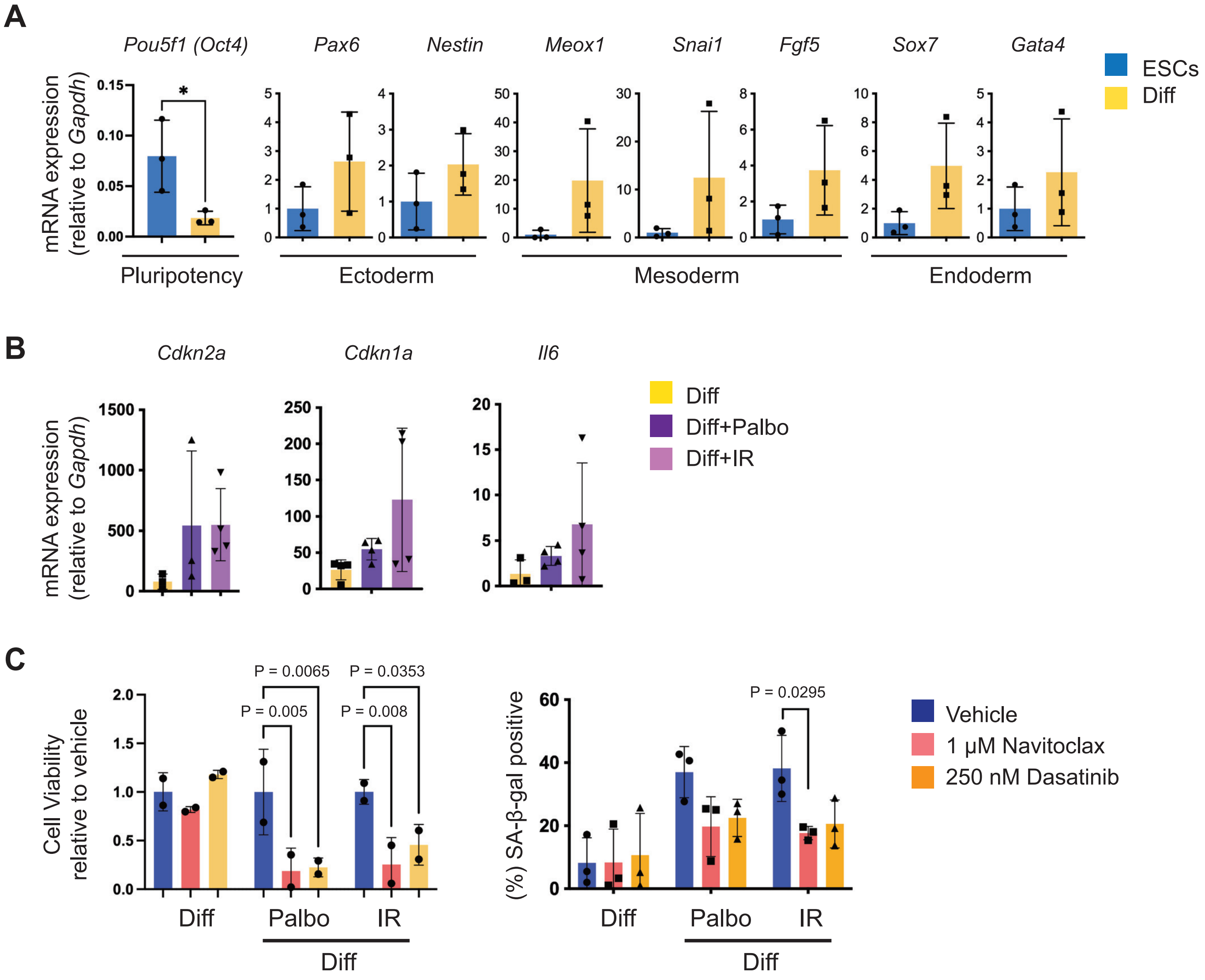

Figure EV1. Validation of the mESC CRISPR/Cas9 screening platform used to identify senolytic targets. Related to Fig. 1.

(**A**) Relative mRNA expression of markers of ES cells (*Pou5f1/Oct4*) and all three germ layers in cells after differentiation induction (Ectoderm= *Pax6, Nestin*; Mesoderm= *Meox1, Snai1, Fgf5*; Endoderm= *Sox7, Gata4*). Signals were normalized to that of *Gapdh*. $n = 3$ biological replicates. (**B**) Relative mRNA expression of senescence markers (*Cdkn2a, Cdkn1A, Il6*) in differentiated cells and cells induced to senescence by Palbociclib or irradiation treatment. Signals were normalized to that of GAPDH. $n = 3$–4 biological replicates. (**C**) Quantification of cell viability and senescence-associated beta-galactosidase (SA-β-gal +) in differentiated and senescent cells, untreated or after 1 μM navitoclax or 250 nM Dasatinib treatment. $n = 3$ biological replicates. Data shown are mean ± SD. Statistical analyses were performed with 2-way ANOVA multiple comparison with Tukey's correction. *P* values are indicated in the figure.

 © The Author(s)

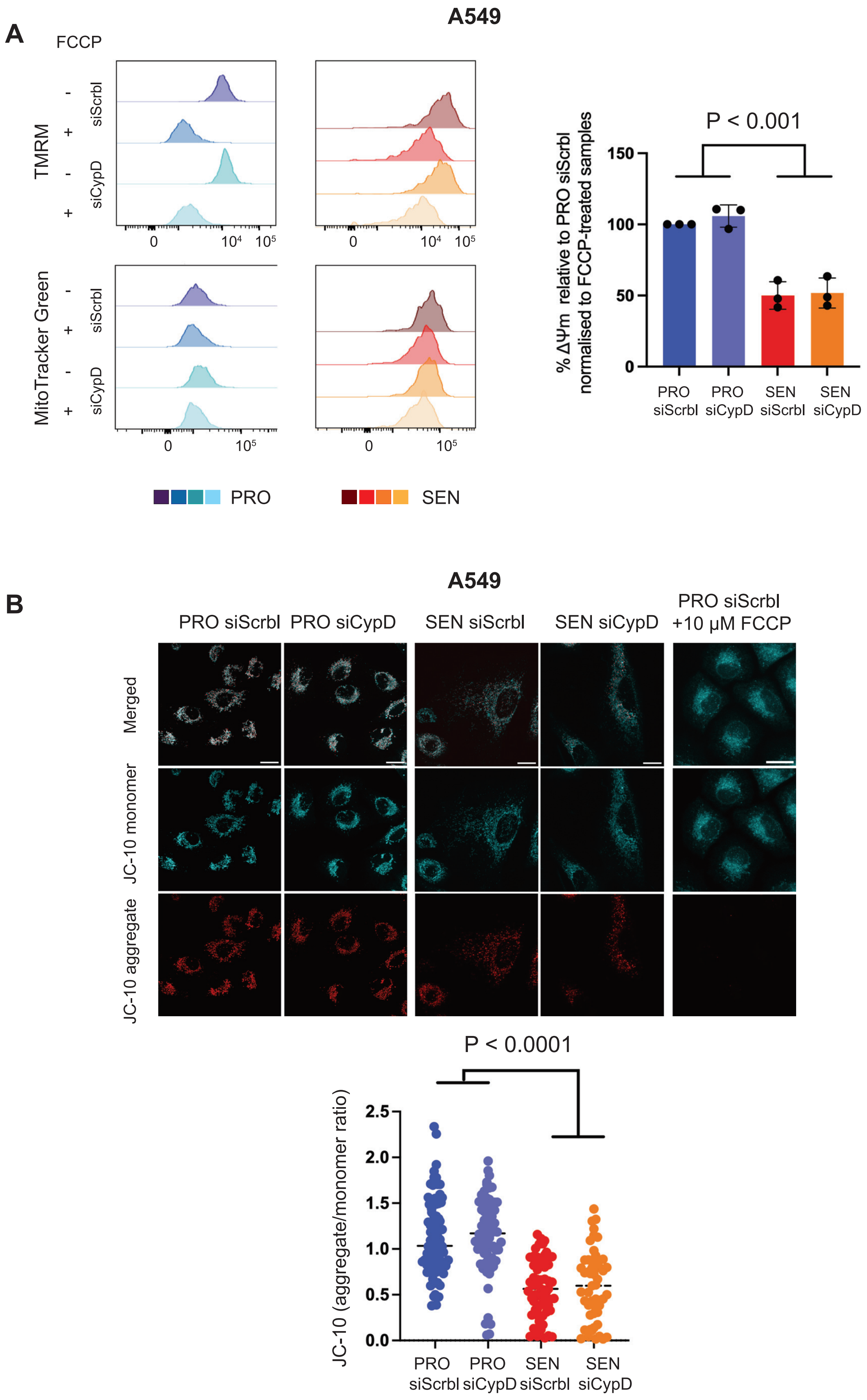
A
A549
FCCP
TMRM
MitoTracker Green
siScrbl
siCypD
0
10⁴
10⁵
PRO
SEN
P < 0.001
% ΔΨm relative to PRO siScrbl normalised to FCCP-treated samples
150
100
50
PRO siScrbl
PRO siCypD
SEN siScrbl
SEN siCypD
B
A549
PRO siScrbl
PRO siCypD
SEN siScrbl
SEN siCypD
PRO siScrbl +10 µM FCCP
Merged
JC-10 monomer
JC-10 aggregate
P < 0.0001
JC-10 (aggregate/monomer ratio)
2.5
2.0
1.5
1.0
0.5
0.0

◀ **Figure EV2. Mitochondrial membrane potential (ΔΨm) is not affected by cyclophilin D depletion. Related to Figs. 4–6.**

(**A**) Representative flow cytometry plot and quantifications of proliferating (blue) and senescent (red) A549 cells, treated with siRNA Scrbl or siRNA against CypD for 5 days, as indicated. Senescence was induced with bleomycin. ΔΨm was measured staining the cells with TMRM. Signal was then normalized to mitochondrial mass, measured co-staining the cells with Mitotracker green. Background fluorescence was assessed in the same samples after treatment with FCCP. $n = 3$ independent experiments. (**B**) Representative images and quantification of JC-10 fluorescence levels in proliferating (blue) senescent (red) A549 cells, 5 days after treatment with siRNA against CypD or siRNA Scrbl. Results are shown as the ratio between red (aggregate) and green (monomer, represented in cyan) signals. $n = 53–85$ cells from a total of 3 independent experiments. The values plotted in the graphs are the mean ± SD. Statistical analyses were performed with 2-way ANOVA multiple comparison with Tukey's correction. *P* values are indicated in the figure.

 © The Author(s)

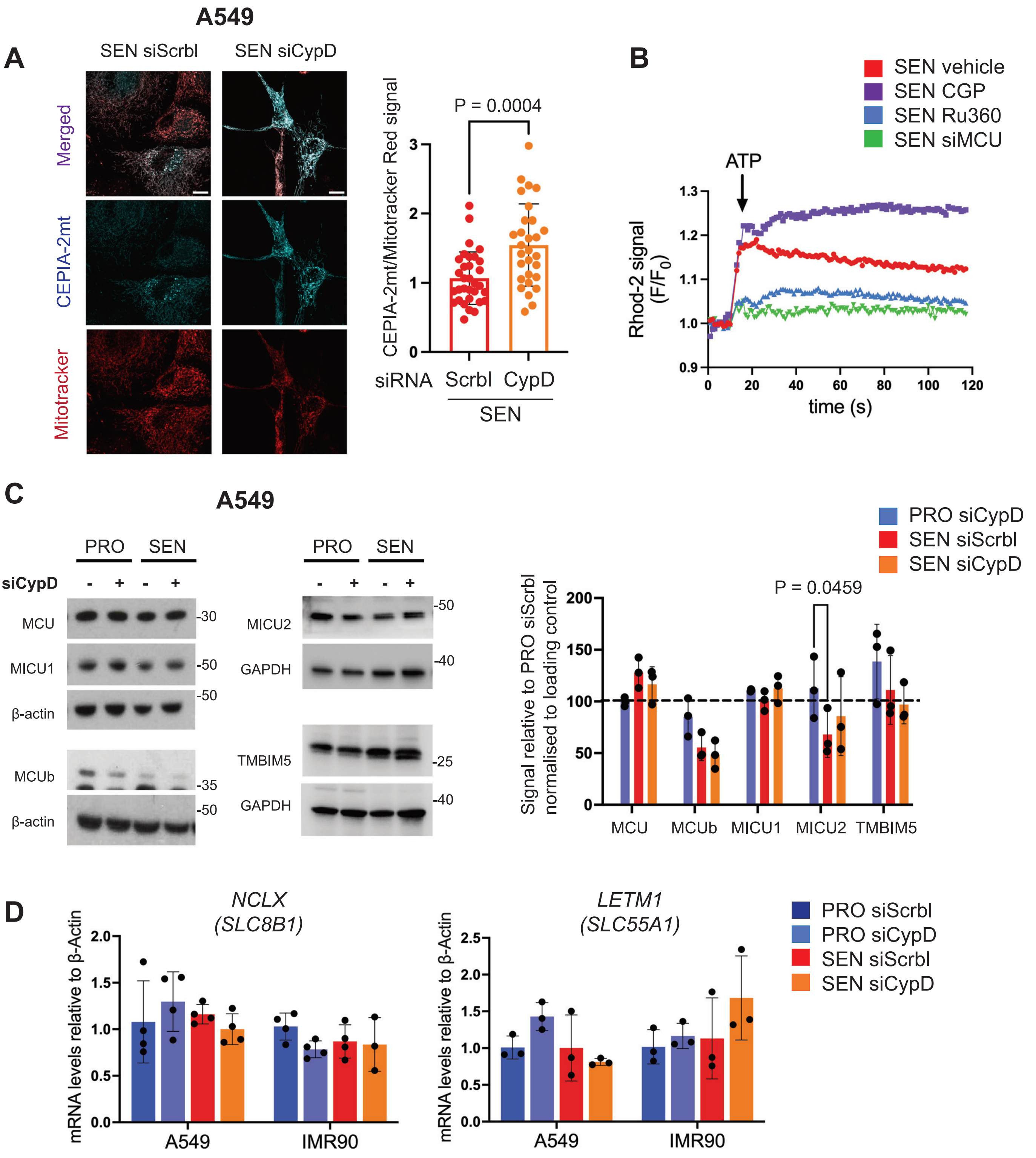
A549
A
SEN siScrbl
SEN siCypD
Merged
CEPIA-2mt
Mitotracker
CEPIA-2mt/Mitotracker Red signal
P = 0.0004
siRNA Scrbl CypD
SEN
B
SEN vehicle
SEN CGP
SEN Ru360
SEN siMCU
ATP
Rhod-2 signal (F/F0)
time (s)
C
A549
PRO SEN
siCypD
MCU
MICU1
β-actin
MCUb
MICU2
GAPDH
TMBIM5
PRO siCypD
SEN siScrbl
SEN siCypD
P = 0.0459
Signal relative to PRO siScrbl normalised to loading control
MCU MCUb MICU1 MICU2 TMBIM5
D
NCLX (SLC8B1)
LETM1 (SLC55A1)
mRNA levels relative to β-Actin
A549 IMR90
PRO siScrbl
PRO siCypD
SEN siScrbl
SEN siCypD

Figure EV3. Mitochondrial calcium imbalances are not due to alterations in transporters expression. Related to Fig. 4.

(**A**) Representative images and quantification of mitochondrial matrix Ca^{2+} levels measured by CEPIA-2mt fluorescent levels in senescent A549 cells 4 days after treatment with siRNA against CypD or siRNA Scrbl. Live-cell images were acquired of cells simultaneously expressing CEPIA-2mt and stained with Mitotracker red. $n = 28$–32 cells from a total of 3 independent experiments. CEPIA-2mt signal was normalized to mitochondrial mass (Mitotracker red signal). (**B**) Average Rhod-2 fluorescent trace in A549 senescent cells treated with vehicle (red trace), pre-treated for 30 min with Ru360 (blue trace), CGP (purple trace), or depleted of MCU (7 days siRNA against MCU, green trace) before and after 10 μM ATP stimulus. $n = 4$ independent experiments. (**C**) Immunodetection of MCU, MICU1, MICU2, MCUb, and TMBIM5 by Western blots of total cell lysates separated by SDS–PAGE. The graphs show the densitometric quantification of the signals corresponding to each protein normalized to that of the loading control, from 3 independent biological replicates of A549 cells. (**D**) Relative mRNA expression of NCLX and LETM1 in proliferating (blue) or senescent (red) A549 and IMR90 cells, treated with siRNA Scrbl or siRNA against CypD for 5 days. Signals were normalized to that of β-actin. $n = 3$–4 biologically independent samples. All the values plotted in the graphs are the mean ± SD. Statistical analyses were performed with 2-way ANOVA multiple comparison with Tukey's correction. *P* values are indicated in the figure.

 © The Author(s)

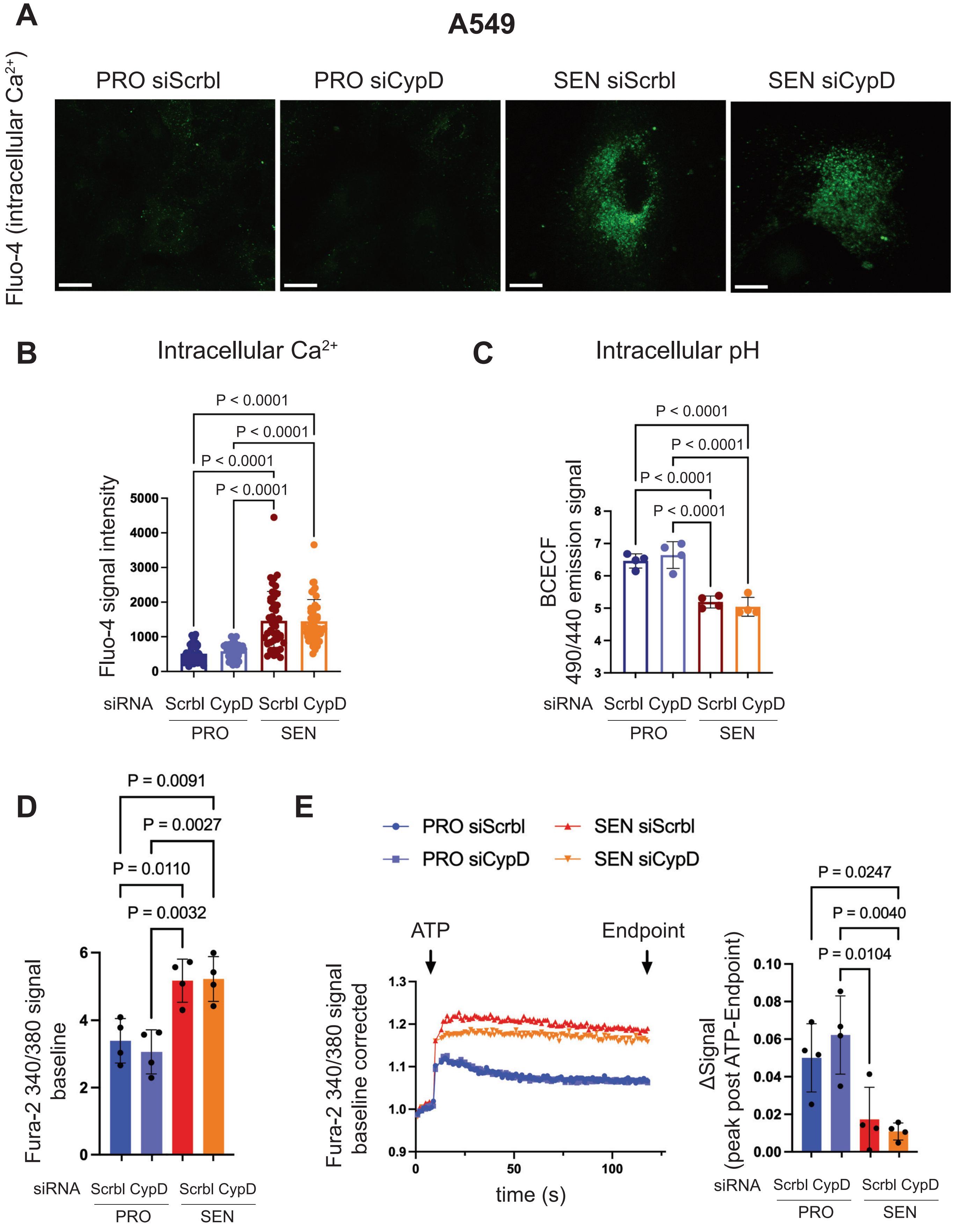
A
A549
Fluo-4 (intracellular Ca2+)
PRO siScrbl
PRO siCypD
SEN siScrbl
SEN siCypD
B
Intracellular Ca2+
P < 0.0001
P < 0.0001
P < 0.0001
P < 0.0001
Fluo-4 signal intensity
5000
4000
3000
2000
1000
0
siRNA
Scrbl CypD Scrbl CypD
PRO
SEN
C
Intracellular pH
P < 0.0001
P < 0.0001
P < 0.0001
P < 0.0001
BCECF 490/440 emission signal
8
7
6
5
4
3
siRNA
Scrbl CypD Scrbl CypD
PRO
SEN
D
P = 0.0091
P = 0.0027
P = 0.0110
P = 0.0032
Fura-2 340/380 signal baseline
6
4
2
0
siRNA
Scrbl CypD Scrbl CypD
PRO
SEN
E
PRO siScrbl
PRO siCypD
SEN siScrbl
SEN siCypD
ATP
Endpoint
Fura-2 340/380 signal baseline corrected
1.3
1.2
1.1
1.0
0.9
0
50
100
time (s)
P = 0.0247
P = 0.0040
P = 0.0104
ΔSignal (peak post ATP-Endpoint)
0.10
0.08
0.06
0.04
0.02
0.00
siRNA
Scrbl CypD Scrbl CypD
PRO
SEN

Figure EV4. Cytosolic calcium and pH are not affected by cyclophilin D depletion. Related to Fig. 4.

(**A**) Representative images of cytosolic calcium levels in proliferating and senescent A549 cells, 5 days after treatment with siRNA against CypD or siRNA Scrbl. Live-cell images were acquired of cells stained with Fluo-4 (20 z-stacks). Scale bar = 20 µm. (**B**) Quantification of Fluo-4 levels from (**A**). $n = 44$–50 cells from a total of 3 independent experiments. (**C**) Quantification of BCECF signals in proliferating (blue) and senescent (red) A549 cells, 5 days after treatment with siRNA against CypD or siRNA Scrbl. $n = 4$ independent experiments. (**D**) Baseline averages of the traces shown in (**E**). Baseline for each experiment is the average of the first 10 s of measurement, before the ATP injection. $n = 4$ independent experiments. (**E**) ATP-induced intracellular Ca^{2+} traces (F340/380) and slopes in proliferating (blue) and senescent (red) A549, expressing or depleted of CypD. Cells were loaded with Fura-2 and stimulated with a single injection of 10 µM ATP. Fluorescence signal was recorded every second for 2 min in total. The traces show the average of $n = 4$ independent experiments. Changes in intracellular Ca^{2+} after the stimulus were quantified as the difference between the maximal fluorescence signal measured post ATP injection (peak) and at endpoint (Δ Signal). All the values plotted in the graphs are the mean ± SD. Statistical analyses were performed with 2-way ANOVA multiple comparison with Tukey's correction. *P* values are indicated in the figure.

 © The Author(s)

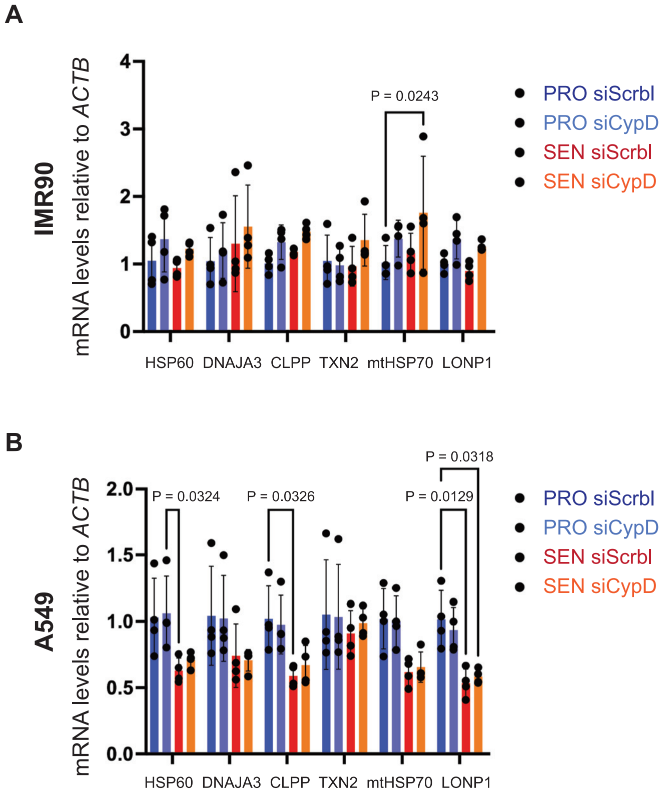

Figure EV5. Cyclophilin D knockdown does not result in UPRmt-associated transcriptional changes. Related to Fig. 5.

Relative mRNA expression of UPRmt markers in proliferating (blue) or senescent (red) IMR90 (**A**) and A549 (**B**) cells, treated with siRNA Scrbl or siRNA against CypD for 5 days. Signals were normalized to that of *β-actin*. $n = 3$–4 biologically independent samples. All the values plotted in the graphs are the mean ± SD. Statistical analyses were performed with 2-way ANOVA multiple comparison with Tukey's correction. *P* values are indicated in the figure.
